# A Novel Strategy for Rapid Fluorescence Detection of FluB and SARS-CoV-2

**DOI:** 10.3390/molecules28052104

**Published:** 2023-02-23

**Authors:** Zhijin Yang, Zhiwei Xue, Kejie Zheng, Yule Zhang, Guorong Sui, Haima Yang, Songlin Zhuang, Lulu Zheng, Dawei Zhang

**Affiliations:** 1 Engineering Research Center of Optical Instrument and System, The Ministry of Education, Shanghai Key Laboratory of Modern Optical System, University of Shanghai for Science and Technology, Shanghai 200093, China; 15195241112@163.com (Z.Y.); jsycxzw@163.com (Z.X.); 18758292095@163.com (K.Z.); zhangyule2017@163.com (Y.Z.); suigr@usst.edu.cn (G.S.); snowyhm@usst.edu.cn (H.Y.); slzhuang@yahoo.com (S.Z.); dwzhang@usst.edu.cn (D.Z.); 2 Shanghai Environmental Biosafety Instruments and Equipment Engineering Technology Research Center, University of Shanghai for Science and Technology, Shanghai 200093, China; 3 Shanghai Institute of Intelligent Science and Technology, Tongji University, Shanghai 200092, China

**Keywords:** SARS-CoV-2, FluB, immunochromatographic test strip, rapid fluorescence detection

## Abstract

Undoubtedly, SARS-CoV-2 has caused an outbreak of pneumonia that evolved into a worldwide pandemic. The confusion of early symptoms of the SARS-CoV-2 infection with other respiratory virus infections made it very difficult to block its spread, leading to the expansion of the outbreak and an unreasonable demand for medical resource allocation. The traditional immunochromatographic test strip (ICTS) can detect one analyte with one sample. Herein, this study presents a novel strategy for the simultaneous rapid detection of FluB/SARS-CoV-2, including quantum dot fluorescent microspheres (QDFM) ICTS and a supporting device. The ICTS could be applied to realize simultaneous detection of FluB and SARS-CoV-2 with one test in a short time. A device supporting FluB/SARS-CoV-2 QDFM ICTS was designed and had the characteristics of being safe, portable, low-cost, relatively stable, and easy to use, ensuring the device could replace the immunofluorescence analyzer in cases where there is no need for quantification. This device does not need to be operated by professional and technical personnel and has commercial application potential.

## 1. Introduction

The severe acute respiratory syndrome coronavirus type 2 (SARS-CoV-2) can be transmitted through aerosols and easily infect humans [1], thus causing the outbreak of pneumonia which evolved into a worldwide pandemic [2]. The early symptoms of SARS-CoV-2 infection and other respiratory viruses, such as influenza B virus (FluB) and influenza A virus (FluA), are similar, with fever and cough. This has led to delays in clinical treatment and made it difficult to block the spread of the disease, furthermore, causing an unreasonable demand for the allocation of medical resources. As a matter of course, useful methods that can distinguish between different viruses, especially SARS-CoV-2 and other respiratory viruses, are urgently needed.

To date, various techniques for the detection of SARS-CoV-2, including real-time reverse transcription-polymerase chain reaction (RT-PCR), enzyme-linked immunosorbent assay (ELISA), and so on [3], have been widely applied in practice and exhibit good accuracy. Nevertheless, the cumbersome and professional operating process brought many difficulties to the large-scale use of these methods, especially during the outbreak. Meanwhile, these technologies take several hours to obtain results which severely limits the speed of detection. Moreover, sampling might increase the risk of virus infection through close person-to-person contact [4]. Thus, there is a high demand for distinguishing between SARS-CoV-2 and other common viruses at home in a short time. Immunochromatographic test strips (ICTS) have been widely accepted as possessing outstanding advantages, including low cost, speed, portability, and user-friendliness [5,6,7,8], and have become a powerful tool for clinical diagnosis. Hence, ICTS are considered a good option to rapidly distinguish different viruses.

One advantage of immunochromatography is the high specificity of antigen–antibody interaction to capture target molecules. Spike protein (S), nucleocapsid protein (N), membrane protein (M), and envelope protein (E) are four structural proteins found in SARS-CoV-2 [9]. Of these, the N protein became an excellent candidate due to its exposure in the process of virus assembly [10]. The N protein also has an early diagnostic value and shortens the window of serological diagnosis [11]. Otherwise, we chose FluB as a representation of common respiratory viruses to construct a system for distinguishing SARS-CoV-2 and other common viruses. The antigens were captured by specific antibodies which is the basis of the method called double-antibody sandwiched antigens. The SARS-CoV-2 N protein-detecting antibody and FluB-detecting antibody were labeled with carboxyl group functionalized quantum dot fluorescent microspheres (QDFM) considering the fluorescence quantum yield and photobleaching ability. Due to the advantages of the fluorescence technique, it has been widely applied in the field of medical detection [12,13]. In addition, many kinds of platforms integrating multi-technology, including microfluidics, were designed and developed for fluorescence detection [14,15].

Traditionally, an immunofluorescence analyzer is applied to analyze the results of fluorescent ICTS and can realize quantitative analysis. However, the price and size of this product limit the range of its application. While a portable flashlight could conveniently provide the excitation light, nevertheless, the results could not be recorded and might be influenced by the surrounding environment. Thus, there is a reasonable demand for the development of a low-cost, portable, relatively stable strategy that could easily record the results of ordinary people in different situations such as at home.

Herein, a fluorescent ICTS based on quantum dot fluorescent microspheres for the point-of-care detection of FluB and SARS-CoV-2 was developed and evaluated. Meanwhile, a low-cost, relatively stable, small, and easy-to-use device that is suitable for smartphones was designed for capturing the pictures of detection results to be uploaded. This is a novel strategy for the rapid detection of FluB and SARS-CoV-2 in many situations which can reduce the risk of infection during sampling, and can be used by anyone to capture pictures using a smartphone without ambient light interference.

## 2. Results

### 2.1. Characterization of QDFM

**Figure 1:**
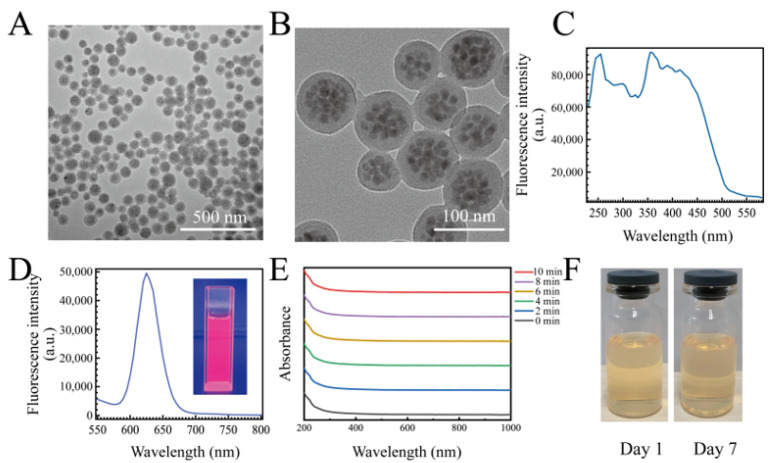
The characterization of the QDFM. (**A**,**B**) TEM images of the QDFM. (**C**,**D**) The excitation and emission spectrum of the QDFM. (**E**) The UV–vis absorbance of QDFM in different time nodes during the UV light exposure. (**F**) The picture of the QDFM suspension on day 1 and day 7.

Before preparing the QDFM-mAb complex, the characterization of the QDFM was significant for ICTS application. The morphology of the QDFM was characterized using a transmission electron microscope (TEM). It is evident from the TEM image that the QDFM consisted of many quantum dots. The average size of the QDFM was also around 100 nm (Figure 1A,B). To choose a suitable light source for exciting the QDFM, an excitation scan was conducted using a multimode microplate reader platform with an emission wavelength of 625 nm. It was obvious that the QDFM could be excited by ultraviolet light including the wavelength of 365 nm (Figure 1C). Meanwhile, the fluorescence emission of the QDFM was analyzed using a microplate reader with 365 nm light excitation. The emission peak of the QDFM appeared at about 625 nm (Figure 1D). The quantum yield of the QDFM was 56.42% (Figure S1 (Supplementary Materials)), measured with a fluorescence spectrophotometer. Thus, the red light of the QDFM was easily observed upon excitation with UV light. In addition, the QDFM had a negligible change in absorption spectrum after UV light exposure, which indicated that the QDFM possessed good photostability (Figure 1E). In addition, the QDFM suspension had no obvious change in color and precipitation on day 1 and day 7 (Figure 1F), suggesting the excellent colloidal stability of the suspension. These standard parameters provided powerful evidence that the QDFM could be employed in the fluorescent ICTS observed by the naked eye.

### 2.2. Usage of mAb

**Figure 2:**
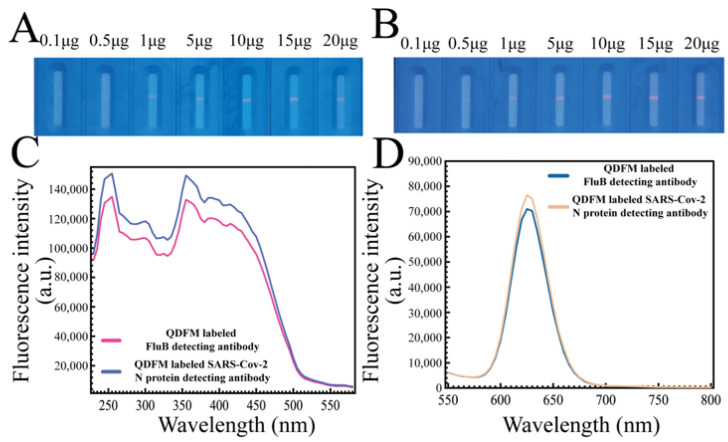
Antibody usage optimization and the characterization of QDFM-mAb conjugation. (**A**,**B**) The fluorescence images of IgG-captured QDFM-mAb conjugation (A. FluB, B. SARS-CoV-2). (**C**,**D**) The excitation and emission spectrum of two kinds of QDFM-mAb conjugation.

The usage of mAb usually plays a key role in the QDFM-mAb complex preparation. When the concentration of mAb is low, the efficiency of the QDFM-mAb conjugation is low. When the amount of mAb is sufficient, the antibody could be easily bound to the QDFM for an increase in collision probability. The optimization of antibody usage became crucial considering the result and cost. The IgG line was used to ensure the amount of mAb in the QDFM-mAb complex preparation because both kinds of detecting antibodies were mouse monoclonal antibodies. For the FluB-detecting antibody, the fluorescence intensity in line C increased with the increase in antibody usage when the amount was small and tended to be relatively stable when the usage ranged from 10 μg to 20 μg (Figure 2A). For the SARS-CoV-2 N protein-detecting antibody, the result was similar to the FluB-detecting antibody (Figure 2B). These results indicate that the QDFM could be successfully labeled with mAb. Herein, 10 μg of mAb (SARS-CoV-2 N protein-detecting antibody or FluB-detecting antibody) was chosen for the preparation of the QDFM-mAb complex considering the balance between effectiveness and cost.

To explore whether the mAb conjugated to QDFM would change the excitation and emission wavelength of the QDFM, the microplate reader platform was applied to analyze the QDFM fluorescence excitation and emission again (Figure 2C,D). The results show that mAb labeling had no significant influence on the excitation and emission peak. Thus, for the QDFM-mAb complex, the excitation light could use light with a wavelength of 365 nm.

### 2.3. Characterization FluB/SARS-CoV-2 ICTS

**Figure 3:**
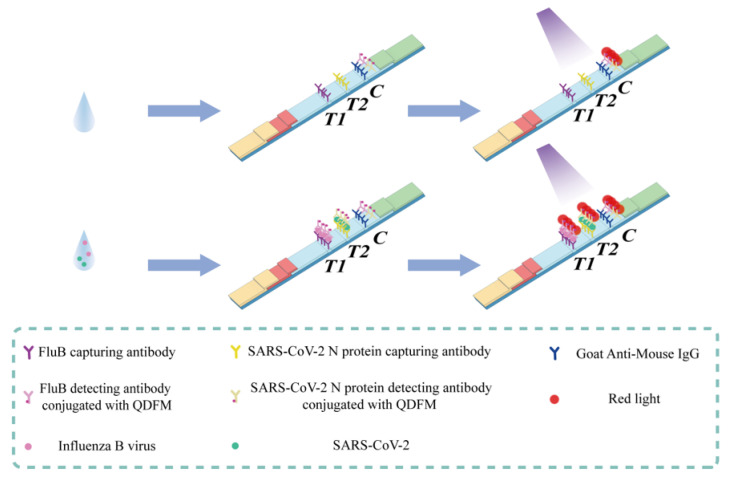
Illustration of the FluB/SARS-CoV-2 ICTS.

The FluB/SARS-CoV-2 ICTS was developed to simultaneously detect SARS-CoV-2 and FluB viruses using one sample (Figure 3). The structure was simple for mass production, with the sample pad, conjugated pad, NC membrane, absorbent pad, and PVC bottom plate. Three different lines were used to connect with FluB, SARS-CoV-2, and the QDFM-mAb complex. When a sample was dripped on the FluB/SARS-CoV-2 ICTS, the flow moved from the sample pad to the absorbent pad with the effect of chromatography. Thus, the flow would cause the movement of the QDFM-mAb complex and lead to antigen-antibody interaction in a short time. When no target virus was present in the sample, the QDFM-mAb complex could not be captured by the FluB-capturing antibody or the SARS-CoV-2 N protein-capturing antibody at line T1 or line T2, and finally ended up at line C for capture by the goat anti-mouse IgG. Under ultraviolet light with a wavelength of 365 nm, line C would appear red due to the excitation of the QDFM. When both targets were in the sample, the QDFM-mAb complex would be captured at lines T1 and T2, and at line C for the excess of the QDFM-mAb complex. Hence, under ultraviolet light, lines T1, T2, and C would appear red because of the excitation of the QDFM.

**Figure 4:**
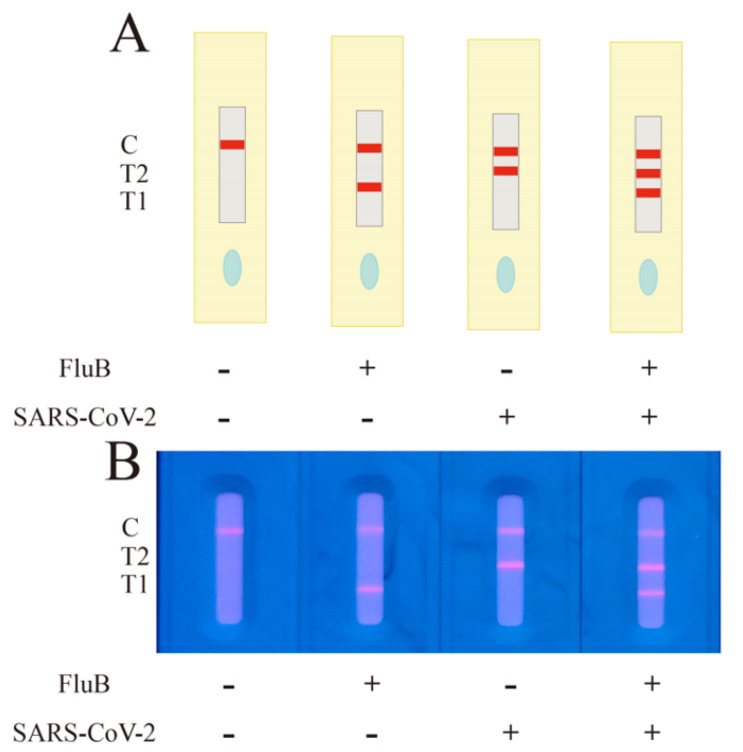
Interpretation of different testing results. (**A**) The four possible situations of detection using FluB/SARS-CoV-2 ICTS in theory. (**B**) The picture of four test results using FluB/SARS-CoV-2 ICTS. “T1”, “T2”, and “C” refer to the lines T1, T2, and C, respectively. The image was captured with a smartphone.

Considering all the situations, four results will be discussed. Two of these cases had no virus present in the sample, and the two target viruses present in the sample have already been mentioned. When only FluB was present in the sample, the QDFM-labeled FluB detecting antibody would interact with FluB to form a new complex that could be captured by the FluB capturing antibody and indicate the existence of different binding sites on the antigen. In this case, under ultraviolet light, lines T1 and C would appear red. When only SARS-CoV-2 was present, the QDFM-labeled SARS-CoV-2 N protein detecting antibody would interact with the target virus to form a new complex that could be captured by its capturing antibody and indicate the existence of different binding sites on the protein. Thus, in this case, line T2 and line C would appear red under ultraviolet light (Figure 4A). The FluB recombinant antigen and SARS-CoV-2 N protein antigen were applied to verify the above inference (Figure 4B). The experiment was consistent with the theory mentioned above.

### 2.4. Detection Performance of the FluB/SARS-CoV-2 ICTS

**Figure 5:**
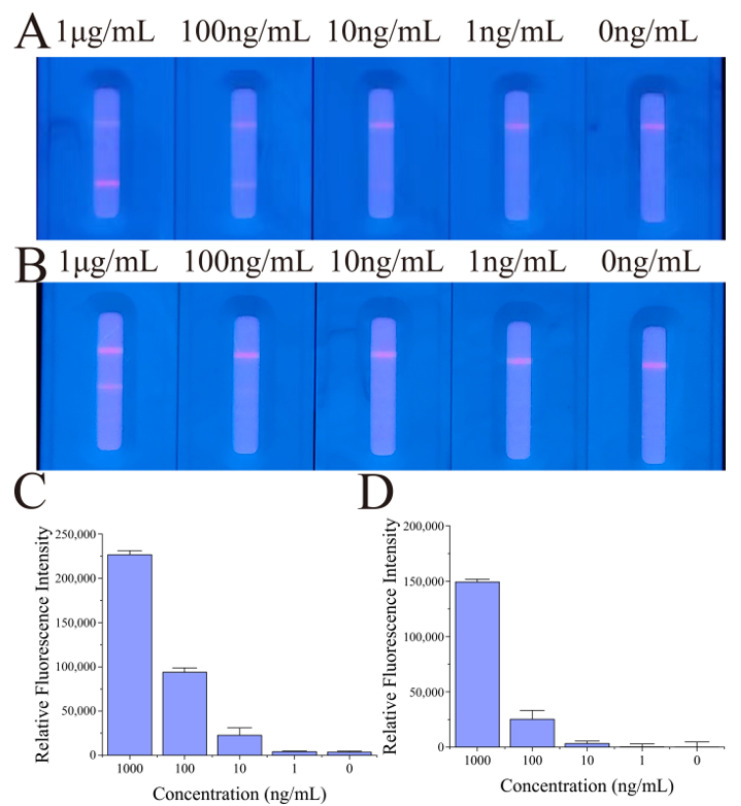
The performance of the FluB/SARS-CoV-2 QDFM ICTS. (**A**) The test of different concentrations of FluB recombinant antigen. (**B**) The test of different concentrations of SARS-CoV-2 N protein antigen. (**C**) The fluorescence analysis of different concentrations of FluB recombinant antigen. (**D**) The fluorescence analysis of different concentrations of SARS-CoV-2 N protein antigen. The images were captured with a smartphone. The analyses were performed using ImageJ.

After antibody usage optimization, the performance of the FluB/SARS-CoV-2 ICTS was evaluated by detecting the different concentrations of FluB recombinant antigen and SARS-CoV-2 N protein antigen. As exhibited in the fluorescence image of the FluB/SARS-CoV-2 ICTS, the red fluorescence became increasingly bright with the increasing concentration of FluB recombinant antigen and SARS-CoV-2 N protein antigen. The fluorescence line T1 for 10 ng/mL of FluB recombinant antigen was distinguished from 0 ng/mL by the naked eye (Figure 5A,C). For the SARS-CoV-2 N protein antigen, 100 ng/mL of the sample could be distinguished from the negative control by the naked eye (Figure 5B,D).

### 2.5. Cross-Reactivity of FluB/SARS-CoV-2 QDFM ICTS

**Figure 6:**
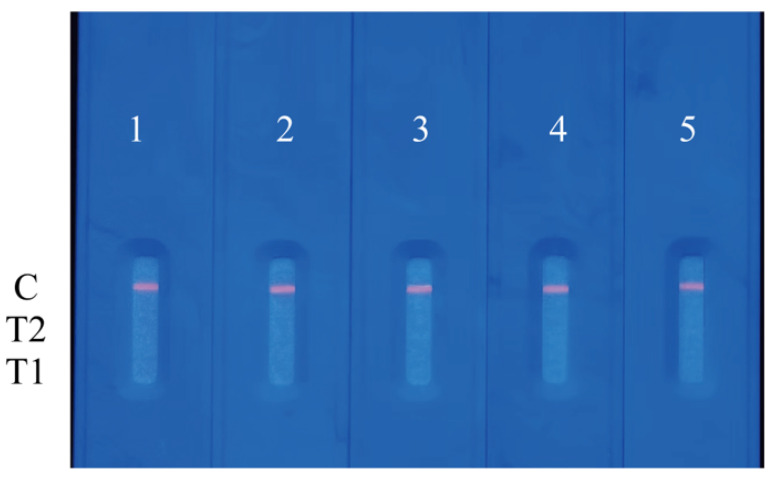
The cross-reactivity of FluB/SARS-CoV-2 QDFM ICTS and antigens of other pathogenic microbes. 1: blank; 2: the antigen of FluA; 3: the antigen of *M.tuberculosis*; 4: the antigen of Escherichia coli; 5: the antigen of salmonella typhi.

The cross-reactivity of FluB/SARS-CoV-2 QDFM ICTS plays a key role in real-world usage, thus cross-reactivity with the antigen of other pathogenic microbes including viruses and bacteria was performed. The antigen of FluA, the antigen of *M.tuberculosis*, the antigen of Escherichia coli, and the antigen of salmonella typhi were used for the cross-reactivity of the FluB/SARS-CoV-2 QDFM ICTS. It was indicated that cross-reactivity among the FluB/SARS-CoV-2 QDFM ICTS and other antigens of pathogenic microbes was not found (Figure 6).

### 2.6. Situation of the Device Suitable for FluB/SARS-CoV-2 QDFM ICTS

**Figure 7:**
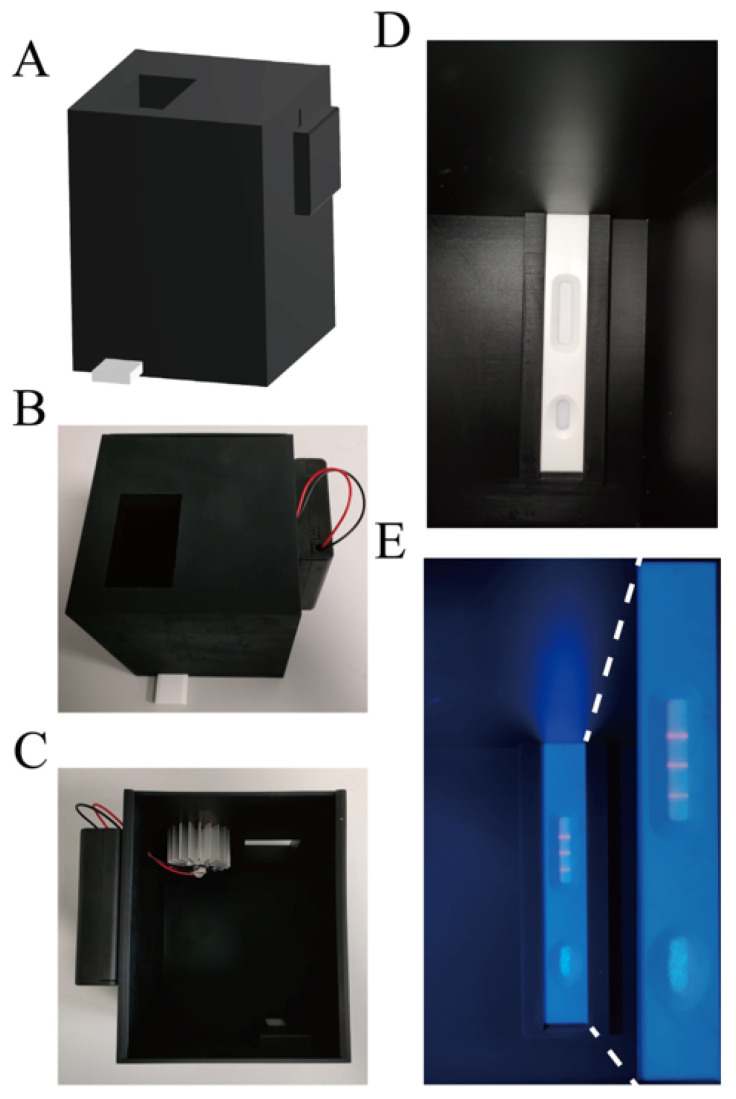
The device supports the FluB/SARS-CoV-2 QDFM ICTS. (**A**,**B**) Structure of the device. (**C**) The position of the ultraviolet LED inside the device. (**D**,**E**) The application of the device.

From a structural perspective, the basic structure of the device was composed of the main body and a removable back plate. The device was made of black resin that could effectively block outside light. The function of the small device (length: 100 mm; width: 100 mm; height: 120 mm) was to provide the excitation light for the QDFM ICTS and keep the space free from ambient light in order to capture pictures with a smartphone. There was a tunnel with a length of 80 mm in the main body in which to place the FluB/SARS-CoV-2 QDFM ICTS. On the top of the main body was a window whose size and position were dependent on the model of the smartphone used. In this work, the window was rectangular with a length of 50 mm and a width of 30 mm (Figure 7A,B). The ultraviolet LED with a cooling machine was placed in the main body, and the battery was placed outside of the main body. The position of the ultraviolet LED was fixed at the top inside the device, with a cooling machine to avoid rapid temperature rises during the recording of the results, which could also extend the service life of this simple device (Figure 7C). The wavelength of the UV LED was 365 nm and the power of the UV LED was 5 W. The circuit of the device was simple, and all components including the power supply, switch, and light source were connected directly, leading to a reduction in operating difficulty. Moreover, the use of fewer components contributes to the portability of the device and ease of replacement of the components.

The cost of the device is much lower than commercial ultraviolet analyzers due to its small size. This low-cost device could also be easily removed due to its size and weight. Significantly, the device is simple for ordinary people to operate, as users only need to place the FluB/SARS-CoV-2 QDFM ICTS into the tunnel of the device, open the switch, and take photographs with a smartphone. The device could be used for capturing both bright fields (the smartphone’s flash acts as a light source) and fluorescence images (light with a wavelength of 365 nm) (Figure 7D,E).

## 3. Discussion

Numerous research groups have reported on methods which have greatly advanced the development of detecting SARS-CoV-2 [3,16,17,18], including RT-PCR and ELISA. Nucleic acid, S protein, IgG, IgM, etc., were common targets for detection in these works. The time of detection in early infection became vital for its infectivity. The convenience of the detection methods is also a significant consideration for large-scale applications due to the current situation of the pandemic. The ICTS, with the characteristics of speed, convenience, low cost, and point-of-care capacity, has been widely accepted across the world in the fields of food, drug testing, etc. [5,8,19,20,21].

The early distinction of the type of virus played a very significant role in reducing patients’ anxiety, saving medical resources, blocking the spread of the SARS-CoV-2 infection, and informing the use of specialized clinical treatment. The special characteristics of the SARS-CoV-2 N protein led it to be chosen as the target for detecting SARS-CoV-2.

Currently, many types of ICTS are usually applied to detect a single molecule, with the shortcoming of being unable to simultaneously detect multiple analytes. The aforementioned ICTS could not meet the requirement of simultaneously distinguishing SARS-CoV-2 from FluB. The design of an ICTS with one test and multiple analytes became a novel strategy to meet the requirement. The FluB/SARS-CoV-2 QDFM ICTS was developed to simultaneously detect two viruses with one test. The QDFM could be separately conjugated with two kinds of detecting antibodies with chemical bonds. In this process, antibody usage optimization was considered. Finally, 10 μg of SARS-CoV-2 N protein-detecting antibody and 10 μg of FluB-detecting antibody were used to form QDFM-mAb conjugation to save costs while providing relatively good results. With assessment, 10 ng/mL of FluB recombinant antigen and 100 ng/mL of SARS-CoV-2 N protein antigen could be distinguished from the negative control by the naked eye, which was similar to previous reports [22,23].

For colloidal gold, the result could be judged by the naked eye without the excitation light. However, the QDFM needed to be excited by the light of a specific wavelength, usually UV light, which could cause damage to the eyes after direct exposure. The immunofluorescence analyzer is widely used in immunochromatography, and is usually found in relevant professional institutions such as hospitals and research institutes. However, the size and weight of the analyzer limits its widespread use. To meet the requirement for using the FluB/SARS-CoV-2 QDFM ICTS at home, a small and portable device, such as an ultraviolet analyzer especially designed for QDFM ICTS, was developed with 3D printing technology. The device is easily portable due to its small volume (approximately 1284 cm^3^, including the volume of the battery) and weight (approximately 305 g). Last but not least, ordinary people could easily capture clear fluorescence images through their smartphones using this portable device.

This is a novel strategy for rapidly distinguishing between SARS-CoV-2 and FluB at home. Users only need to dilute the sample, add it to the ICTS, wait for 20 min, put the ICTS into the device, and take the images. These are simple steps that almost anyone can perform. Thus, this strategy might become a new trend for detection at home in the future.

## 4. Materials and Methods

### 4.1. Reagents and Instruments

The bovine serum albumin (BSA) and goat anti-mouse IgG were purchased from Solarbio (Beijing, China). The phosphate buffer saline (PBS) was obtained from Servicebio (Wuhan, China). The N-(3-Dimethylaminopropyl)-N′-ethylcarbodiimide hydrochloride (EDC) and N-Hydroxysulfosuccinimide sodium salt (sulfo-NHS) were supplied by Aladdin (Shanghai, China). The sodium dihydrogen phosphate, disodium hydrogen phosphate, sucrose, 2-Morpholinoethanesulfonic acid (MES), and trehalose were obtained from Sinopharm (Shanghai, China). The carboxyl group functionalized ZnCdSe/ZnS quantum dot fluorescent microspheres were obtained from Jiayuan Quantum Dots Co., Ltd. (Wuhan, China).

The sample pad and conjugated pad were obtained from Jieyi Biotechnology Co., Ltd. (Shanghai, China). The absorbent pad and PVC bottom plate were provided by MICRODETECTION (Nanjing, China). The nitrocellulose (NC) membranes (CN140) were obtained from Sartorius (Shanghai, China).

The equipment used in this work includes a fluorescence spectrophotometer from Edinburgh Instruments (Edinburgh, UK), the ultrapure system from Millipore Co., Ltd. (Bedford, MA, USA), a dispensing system from LRA (Tianjin, China), a high-speed freezing centrifuge from Beckman Coulter (State of California, USA), an ultrasonic apparatus from Kunshan Ultrasonic Instrument Co., Ltd. (Suzhou, China), a multimode microplate reader platform from TECAN (Männedorf, Switzerland), and a smartphone from Realme (Shenzhen, China).

### 4.2. Measurement of Quantum Yield

The quantum yield was measured using a fluorescence spectrophotometer, and the absolute quantum yield was obtained using an integrating sphere.

### 4.3. Photo and Colloidal Stability

The change in the UV-vis absorption spectrum became a powerful tool for evaluating photostability. To test the photostability of the QDFM, UV light was applied to expose the QDFM for a period of time. During the exposure to UV light, the UV-vis absorption spectrum was tested at different time nodes.

To ensure the colloidal stability of the QDFM suspension, the QDFM was dispersed into water. On day 1 and day 7, the QDFM suspension was recorded using the smartphone, and the colloidal stability was judged by the change in color and precipitation.

### 4.4. Preparation of QDFM-Monoclonal Antibody (mAb) Complex

The amino groups in the SARS-CoV-2 N protein-detecting antibody and FluB-detecting antibody could be connected with the carboxyl group of QDFM. The antibodies were conjugated with QDFM according to the protocol described by Liang with slight modifications [24]. The 10 μL of QDFM was washed once with MES solution (pH 6.0, 25 mM), collected by centrifugation (15,000 rpm, 10 min), and resuspended in 100 μL of MES solution. The suspension was ultrasonicated for the uniform dispersion of QDFM. Then, the freshly prepared EDC (10 mg/mL, 2 μL) and sulfo-NHS (10 mg/mL, 5 μL) were added to the suspension, fully mixed, and left for 20 min at 25 °C. Subsequently, the activated QDFM were collected by centrifugation and redispersed in 1 mL PB solution (10 mM, pH 7.7). The SARS-CoV-2 N protein-detecting antibody or FluB-detecting antibody was added to the aforementioned solution, then mixed thoroughly. After 1.5 h, 100 μL of 20% BSA (*w/v*) was added into the aforementioned solution for blocking unbound sites for 30 min at a temperature of 25 °C. Following centrifugation at a speed of 15,000 rpm and a time of 8 min, the precipitate was resuspended in 100 μL of buffer (PB buffer, 10 mM, pH 7.4) containing 10% sucrose (*w/v*), 5% trehalose (*w/v*), 1% BSA (*w/v*), and 1% Tween-20, and then stored at 4 °C for use.

### 4.5. Construction of the ICTS

Two kinds of ICTS were developed: one for optimizing antibody usage (the test ICTS), and another for distinguishing between SARS-CoV-2 and FluB (the FluB/SARS-CoV-2 ICTS). The basic structure of an ICTS includes five parts: the sample pad, conjugated pad, NC membrane, absorbent pad, and PVC bottom plate. The only difference between the test ICTS and the FluB/SARS-CoV-2 ICTS was the antibody that was sprayed onto the NC membrane. In the test ICTS, only the goat anti-mouse IgG which was dissolved with PBS buffer (10 mM, pH 7.4) containing 0.1% sucrose (*w/v*) was sprayed onto the NC membrane. In the FluB/SARS-CoV-2 ICTS, the FluB-capturing antibody, the SARS-CoV-2 N protein-capturing antibody, and the IgG were sprayed onto the NC membrane to form line T1, line T2, and line C, respectively. Before these processes, the NC membrane was pasted onto the PVC bottom plate in advance. The sprayed NC membrane was put in a dry box with a temperature of 37 °C for half a day, and then the absorbent pad, the conjugated pad, and the sample pad were pasted onto the PVC bottom plate. After this, the plate was cut into strips with a cutting machine and stored in a dry atmosphere at 37 °C for use.

### 4.6. Antibody Usage

The usage of antibodies played a significant role in the preparation of the QDFM-mAb complex. Different amounts of antibody were added into the suspension containing activated QDFM to optimize the usage of the antibody, all else being the same. The QDFM-mAb complex was diluted and then dripped onto the sample pad. After incubation at a temperature of 25 °C for 20 min, the test strips were put into the dark box ultraviolet analyzer, and pictures were captured using the smartphone.

### 4.7. Test of FluB/SARS-CoV-2 ICTS

The two target testing objects were diluted to different concentrations. The aforementioned solution was mixed with two kinds of QDFM-mAb complex and then dripped onto the sample pad. After incubation, they were put into the ultraviolet analyzer.

### 4.8. Cross-Reactivity of FluB/SARS-CoV-2 QDFM ICTS

Different kinds of testing objects were diluted and then added into the FluB/SARS-CoV-2 QDFM ICTS. After 20 min, the result was recorded using the smartphone. Five groups were set for tests of cross-reactivity, including the blank group, the antigen of the FluA group, the antigen of the *M.tuberculosis* group, the antigen of the Escherichia coli group, and the antigen of the salmonella typhi group. The blank group acted as a negative control.

### 4.9. User-Friendly Device Suitable for the FluB/SARS-CoV-2 ICTS

The device was composed of two main parts: the main body which was equipped with a light source, and a removable back plate which was used for changing the light source when needed. The main part of the device was made with 3D printing technology. Black resin was chosen as the body material with the aim of reducing ambient light interference. The light source was a light-emitting diode (LED) that could emit ultraviolet light (about 365 nm) to excite the QDFM. There was a window on this device for capturing a picture using the smartphone.

## 5. Conclusions

In summary, the novel strategy for the rapid detection of FluB and SARS-CoV-2 using one sample at home could reduce the anxiety of people who have fever and cough, which are early symptoms of SARS-CoV-2 infection. The results can be recorded using a smartphone and uploaded to the system. The FluB/SARS-CoV-2 QDFM ICTS could quickly tell us the results. An amount of 10 ng/mL of FluB recombinant antigen and 100 ng/mL of SARS-CoV-2 N protein antigen could be distinguished from the respective negative control by the naked eye. We believe that the FluB/SARS-CoV-2 QDFM ICTS could realize the higher sensitivity of detection of the two viruses after future optimization, which is our current plan. The device is suitable for FluB/SARS-CoV-2 QDFM ICTS and is safe, portable, low cost, relatively stable, and easy to use, meaning it could replace the immunofluorescence analyzer in cases where there is no need for quantification. This device does not need professional and technical personnel to operate and has commercial application potential.

## Data Availability

Not applicable.

## Supplementary Materials

The following supporting information can be downloaded at: https://www.mdpi.com/article/10.3390/molecules28052104/s1, Figure S1: The quantum yield result of the QDFM.

## References

[1] Liu Y., Ning Z., Chen Y., Guo M., Liu Y., Gali N.K., Sun L., Duan Y., Cai J., Westerdahl D. (2020). Aerodynamic Analysis of SARS-CoV-2 in Two Wuhan Hospitals. Nature.

[2] Baloch S., Baloch M.A., Zheng T., Pei X. (2020). The Coronavirus Disease 2019 (COVID-19) Pandemic. Tohoku J. Exp. Med..

[3] Cui F., Zhou H.S. (2020). Diagnostic Methods and Potential Portable Biosensors for Coronavirus Disease 2019. Biosens. Bioelectron..

[4] Chan J.F.W., Yuan S., Kok K.H., To K.K.W., Chu H., Yang J., Xing F., Liu J., Yip C.C.Y., Poon R.W.S. (2020). A Familial Cluster of Pneumonia Associated with the 2019 Novel Coronavirus Indicating Person-to-Person Transmission: A Study of a Family Cluster. Lancet.

[5] Yang Y.C., Liu M.H., Yang S.M., Chan Y.H. (2021). Bimodal Multiplexed Detection of Tumor Markers in Non-Small Cell Lung Cancer with Polymer Dot-Based Immunoassay. ACS Sens..

[6] Huang X., Aguilar Z.P., Xu H., Lai W., Xiong Y. (2015). Membrane-Based Lateral Flow Immunochromatographic Strip with Nanoparticles as Reporters for Detection: A Review. Biosens. Bioelectron..

[7] Zeng L., Xu X., Guo L., Wang Z., Ding H., Song S., Xu L., Kuang H., Liu L., Xu C. (2021). An Immunochromatographic Sensor for Ultrasensitive and Direct Detection of Histamine in Fish. J. Hazard. Mater..

[8] Wang L., Sun J., Ye J., Wang L., Sun X. (2022). One-Step Extraction and Simultaneous Quantitative Fluorescence Immunochromatography Strip for AFB1 and Cd Detection in Grain. Food Chem..

[9] Boson B., Legros V., Zhou B., Siret E., Mathieu C., Cosset F.L., Lavillette D., Denolly S. (2021). The SARS-CoV-2 Envelope and Membrane Proteins Modulate Maturation and Retention of the Spike Protein, Allowing Assembly of Virus-like Particles. J. Biol. Chem..

[10] Bai C., Zhong Q., Gao G.F. (2022). Overview of SARS-CoV-2 Genome-Encoded Proteins. Sci. China Life Sci..

[11] Li T., Wang L., Wang H., Li X., Zhang S., Xu Y., Wei W. (2020). Serum SARS-CoV-2 Nucleocapsid Protein: A Sensitivity and Specificity Early Diagnostic Marker for SARS-CoV-2 Infection. Front. Cell. Infect. Microbiol..

[12] Natarajan S., DeRosa M.C., Shah M.I., Jayaraj J. (2021). Development and Evaluation of a Quantitative Fluorescent Lateral Flow Immunoassay for Cystatin-C, a Renal Dysfunction Biomarker. Sensors.

[13] Radha R., Shahzadi S.K., Al-Sayah M.H. (2021). Fluorescent Immunoassays for Detection and Quantification of Cardiac Troponin I: A Short Review. Molecules.

[14] Canals J., Franch N., Alonso O., Vilà A., Diéguez A. (2019). A point-of-care device for molecular diagnosis based on CMOS SPAD detectors with integrated microfluidics. Sensors.

[15] Lovecchio N., Costantini F., Parisi E., Nardecchia M., Tucci M., Nascetti A., De Cesare G., Caputo D. (2018). Integrated optoelectronic device for detection of fluorescent molecules. IEEE Trans. Biomed. Circuits Syst..

[16] Chen H., Park S.G., Choi N., Kwon H.J., Kang T., Lee M.K., Choo J. (2021). Sensitive Detection of SARS-CoV-2 Using a SERS-Based Aptasensor. ACS Sens..

[17] Ding X., Yin K., Li Z., Lalla R.V., Ballesteros E., Sfeir M.M., Liu C. (2020). Ultrasensitive and Visual Detection of SARS-CoV-2 Using All-in-One Dual CRISPR-Cas12a Assay. Nat. Commun..

[18] Lin Q., Wen D., Wu J., Liu L., Wu W., Fang X., Kong J. (2020). Microfluidic Immunoassays for Sensitive and Simultaneous Detection of IgG/IgM/Antigen of SARS-CoV-2 within 15 Min. Anal. Chem..

[19] Thongkum W., Hadpech S., Tawon Y., Cressey T.R., Tayapiwatana C. (2019). Semi-Quantification of HIV-1 Protease Inhibitor Concentrations in Clinical Samples of HIV-Infected Patients Using a Gold Nanoparticle-Based Immunochromatographic Assay. Anal. Chim. Acta.

[20] Yao J., Wang Z., Guo L., Xu X., Liu L., Kuang H., Xu C. (2021). Lateral Flow Immunoassay for the Simultaneous Detection of Fipronil and Its Metabolites in Food Samples. Food Chem..

[21] Sun Y., Song S., Wu A., Liu L., Kuang H., Xu C. (2021). A Fluorescent Paper Biosensor for the Rapid and Ultrasensitive Detection of Zearalenone in Corn and Wheat. Anal. Methods.

[22] Zhang C., Zhou L., Du K., Zhang Y., Wang J., Chen L., Lyu Y., Li J., Liu H., Huo J. (2020). Foundation and Clinical Evaluation of a New Method for Detecting SARS-CoV-2 Antigen by Fluorescent Microsphere Immunochromatography. Front. Cell. Infect. Microbiol..

[23] Xie C., Ding H., Ding J., Xue Y., Lu S., Lv H. (2022). Preparation of Highly Specific Monoclonal Antibodies against SARS-CoV-2 Nucleocapsid Protein and the Preliminary Development of Antigen Detection Test Strips. J. Med. Virol..

[24] Liang Z., Peng T., Jiao X., Zhao Y., Xie J., Jiang Y., Meng B., Fang X., Yu X., Dai X. (2022). Latex Microsphere-Based Bicolor Immunochromatography for Qualitative Detection of Neutralizing Antibody against SARS-CoV-2. Biosensor.

