# Supplementary figures and images for: A Novel Strategy for Rapid Fluorescence Detection of FluB and SARS-CoV-2
Source: Molecules. 2023 Feb 23;28(5):2104. doi: 10.3390/molecules28052104 (PMC10004075; doi:10.3390/molecules28052104)

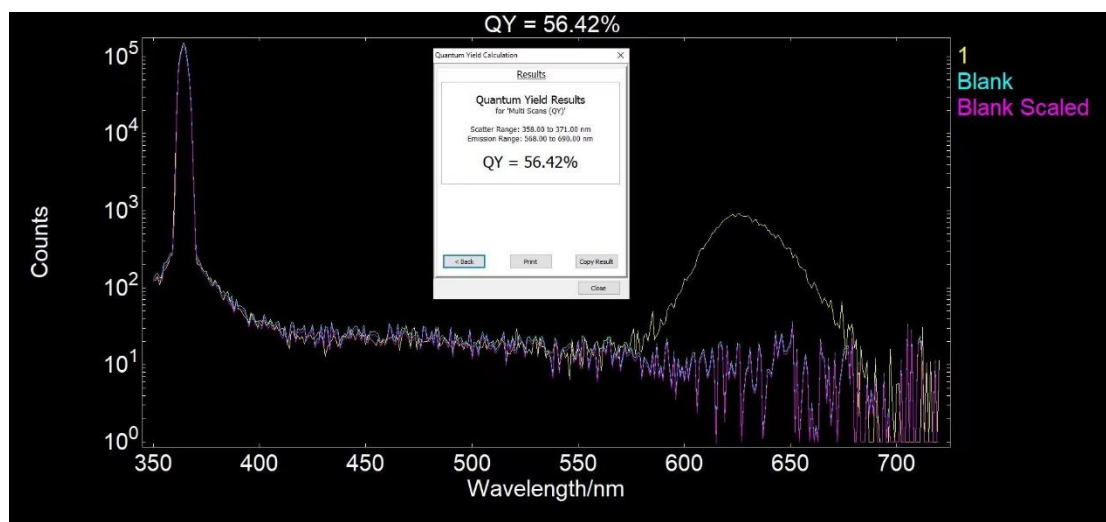

**Figure S1.** The quantum yield result of the QDFM

Supplement: Supplementary file 1 [file molecules-28-02104-s001.zip › molecules-2174950-supplementary.pdf]
